# Isolated follicle stimulated hormone deficiency in male: case report

**DOI:** 10.1186/s13104-017-3109-4

**Published:** 2018-01-15

**Authors:** Gowri M. Ratnayake, P. N. Weerathunga, L. P. Ruwanpura, Amila Wickramasinghe, Prasad Katulanda

**Affiliations:** 1 National Hospital of Sri Lanka, Colombo, Sri Lanka; 2 Colombo South Teaching Hospital, Kalubowila, Sri Lanka; 3 Sri Jayawardenepura General Hospital, Sri Jayawardenepura Kotte, Sri Lanka; 4 Department of Clinical Medicine, University of Colombo, Colombo, Sri Lanka

**Keywords:** Azoospermia, Follicle stimulating hormone, Subfertility

## Abstract

**Background:**

Recent rapid advances in assisted reproductive health technologies enables couples with subfertility to conceive through various intervention. Majority of treatment modalities target the female partner. However it is important to identify and treat male factor subfertility right at the outset. We report a case of isolated follicle stimulating hormone deficiency resulting in azoospermia and primary subfertility.

**Case presentation:**

A 28 year otherwise healthy male presented with primary subfertility with a healthy female counterpart. He was found to have non obstructive azoospermia with low seminal fluid volume. He had normal external genitalia and potency with increased libido. Further evaluation revealed an isolated deficiency of follicle stimulating hormone with elevated testosterone levels. His luteinizing hormone and prolactin levels were normal. Contrast enhanced CT scan of chest, abdomen and pelvis and MRI scan of the pituitary fossa were normal too.

**Conclusion:**

In the era of modern reproductive technology it is important to further evaluate males with non-obstructive azoospermia to detect underlying gonadotropin deficiency.

## Background

Human follicle stimulating hormone (FSH) and luteinizing hormone (LH) are pituitary glycoprotein hormones that regulate gonadal functions in both males and females. In males FSH stimulate sertoli cell proliferation and spermatogenesis, while LH stimulates Leydig cells to produce testosterone. Several studies reported positive outcome of FSH fertilizing on sperm quality and its fertilizing capacity [1, 2] and sperm production [2].

Azoospermia is defined as absence of sperm in the ejaculate and affects 1% of male population and 10–15% of men who seek fertility treatment [3]. Majority (89%) of patients with non-obstructive azoospermia have high FSH levels [4]. However, Isolated FSH deficiency was detected in 0.87% of those with male factor subfertility [5].

## Case presentation

A 28 year old male presented with primary subfertility and increased libido for 8 years. He was an otherwise healthy businessman with a BMI of 22.34 kg/m^2^. He had normal virilization and normal potency. His physical examination including the external genitalia was essentially normal. Evaluation of the partner excluded female factor subfertility.

His seminal fluid analysis revealed azoospermia with low seminal fluid volume, a normal pH and liquefaction time (WHO 2010 criteria). USS scan and the Doppler studies of the scrotum revealed normal testicles with no cause for azoospermia detected. His serum testosterone level was higher than the normal; 42.15 nmol/L (4.94–32.01). Serum FSH levels were 0.56 µIU/mL (0.7–11.1). His serum LH levels, thyroid profile, serum prolactin levels and serum 9 am cortisol levels were all normal. MRI of the pituitary fossa was normal. Contrast enhanced CT scan of the chest, abdomen and pelvis did not demonstrate ectopic testosterone secreting tumors. His basic investigations including blood sugar, serum electrolytes, creatinine and cholesterol levels were normal.

It was planned to start on a course of recombinant subcutaneous FSH injections as the treatment of isolated FSH deficiency.

## Discussion and conclusions

Patient in this case report presented with male factor subfertility due to non-obstructive azoospermia resulting from isolated deficiency of FSH. His increased libido can be explained by the presence of high circulatory levels of testosterone without evidence of a testosterone secreting tumor.

Hypogonadism is main cause of non-obstructive azoospermia; which may be due to hyper gonadotropic hypogonadism (increased FSH and LH with low testosterone) or hypo gonadotrophic hypogonadism (low or inappropriately normal FSH and LH with low testosterone). One of the reasons for azoospermia in patients with hypogonadism is low intra testicular testosterone [3, 4]. However patient in this case report had high endogenous testosterone production with azoospermia with non-suppressed LH levels and low FSH levels.

Reports on isolated FSH deficiency resulting in Male factor subfertility are exceedingly rare with a prevalence of 0.89% in a sample of 3335 of males with subfertility reported from a retrospective study on ‘isolated FSH deficiency in infertile males-preliminary report’ from Egypt based on data collected over a period of 8 years. In this study 29 patients with isolated FSH deficiency were found to have oligoteratoasthenozoospermia rather than azoospermia. Furthermore the most affected sperm parameter was abnormal sperm morphology and those with an abnormal sperm parameters had a low FSH levels as compared to the normal FSH levels detected those with a normal sperm parameters [5].

Reported case reports on isolated FSH deficiency and azoospermia states normal testosterone and LH levels [5, 6]. Furthermore men with non-obstructive azoospermia have high circulatory levels of FSH with low or normal testosterone levels [4]. The patient in this case report with non obstructive azoospermia had low levels of FSH and high levels of testosterone levels. As a result of low FSH level Inhibin production by sertoli cells in the testes is impaired. This results into high levels of GnRH secretion by hypothalamus due to lack of feedback inhibition. High GNRH activation may result in subsequent high LH and testosterone levels. Case study of a patient with isolated FSH deficiency showed unresponsiveness to reported GnRH stimulation and absent mutations in beta-FSH producing gene. However spermatogenesis was successfully induced in a case of isolated FSH deficiency with azoospermia following treatment with human menopausal gonadotropin [6]. Further studies are required to ascertain a specific cause for isolated FSH deficiency in men.

In conclusion males with non-obstructive azoospermia needs further evaluation with hormonal assays to detect gonadotropin deficiency. Because such males may be having elevated levels of testosterone and LH despite markedly low FSH levels.

## Abbreviations

FSH: follicle stimulating hormone
LH: luteinizing hormone
GnRH: gonadotropin releasing hormone
WHO: World Health Organization
CT: computed tomography
